# Selected statins produce rapid spinal motor neuron loss in vitro

**DOI:** 10.1186/1471-2474-13-100

**Published:** 2012-06-15

**Authors:** Beth B Murinson, Norman J Haughey, Nicholas J Maragakis

**Affiliations:** 1 Department of Neurology, Johns Hopkins School of Medicine, Baltimore, USA; 2 Department of Neurology, Rambam Health Care Campus, Haifa, Israel

**Keywords:** ALS, Peripheral neuropathy, Statins, Toxicity, Motorneuronopathy

## Abstract

**Background:**

Hmg-CoA reductase inhibitors (statins) are widely used to prevent disease associated with vascular disease and hyperlipidemia. Although side effects are uncommon, clinical observations suggest statin exposure may exacerbate neuromuscular diseases, including peripheral neuropathy and amyotrophic lateral sclerosis. Although some have postulated class-effects, prior studies of hepatocytes and myocytes indicate that the statins may exhibit differential effects. Studies of neuronal cells have been limited.

**Methods:**

We examined the effects of statins on cultured neurons and Schwann cells. Cultured spinal motor neurons were grown on transwell inserts and assessed for viability using immunochemical staining for SMI-32. Cultured cortical neurons and Schwann cells were assessed using dynamic viability markers.

**Results:**

7 days of exposure to fluvastatin depleted spinal motor neurons in a dose-dependent manner with a K_D_ of < 2 μM. Profound neurite loss was observed after 4 days exposure in culture. Other statins were found to produce toxic effects at much higher concentrations. In contrast, no such toxicity was observed for cultured Schwann cells or cortical neurons.

**Conclusions:**

It is known from pharmacokinetic studies that daily treatment of young adults with fluvastatin can produce serum levels in the single micromolar range. We conclude that specific mechanisms may explain neuromuscular disease worsening with statins and further study is needed.

## Background

Hmg-CoA reductase inhibitors (statins) are widely used for lowering cholesterol and the prevention of cardiovascular and cerebrovascular morbidity and mortality. Indeed, the use of statins has become more prevalent over time and selected populations, such as those discharged after hospital admission for stroke, have prevalence rates for statin prescription exceeding 80% [1].

Side effects of statin treatment are relatively uncommon. Rhabdomyolysis predominates as a side effect that may necessitate treatment cessation [2,3]. Myalgias are more prevalent but often accepted as part of treatment [4]. Laboratory studies to investigate the mechanisms underlying muscle-related effects have shown that statins have negative effects on oxidative phosphorylation by muscle mitochondria [5]. The concentrations at which specific statins produce these effects varies widely and are generally considered to be outside the normal pharmacological dosing range except for cerivistatin (now withdrawn from the market) and possibly fluvastatin [6]. More recent studies have shown that statins alter important second messenger events, e.g., the linkage of proteins to membrane via lipophilic isoprenyl molecules [5] the synthesis of which is directly inhibited by statins [7].

Clinical observations have suggested that statin exposure may unmask or accelerate the course of amyotrophic lateral sclerosis, (ALS) [8]. Substantial controversy has accompanied these observations and based on current studies, it is not clear whether there is a causal association or merely selection bias [9,10]. Selection bias could arise if concerns about statins among ALS patients and their physicians lead to increased reporting of suspected effects. At present several questions remain unanswered. Among these is whether there is evidence that statins are directly toxic to spinal motor neurons. An additional unresolved question regards the role of statins with regards to peripheral neuropathy. Large scale epidemiological studies have suggested that statins are associated with an increased risk for peripheral neuropathy [11,12] but the magnitude and significance of this risk remains unresolved.

The culture of spinal motoneurons in organotypic spinal cord cultures has been an instructive methodology to better understand the neurobiology of the primary motor neurons [13] and has been used as a screening tool for the examination of potential candidate drugs for neuroprotective strategies in ALS [14]. Because of persistent concerns that statins may impact the course of ALS and prompt the development of peripheral neuropathy, we sought to examine the effects of statins on spinal motoneurons in culture.

## Methods

### Spinal motoneuron cultures

All reagents were obtained from Sigma-Aldrich unless otherwise specified. Organotypic spinal cord cultures were prepared under sterile conditions from the lumbar spinal cords of 8-day old rat pups, using an approved protocol as previously described [13]. In brief, transverse 350 mm sections were prepared with a McIlwain tissue chopper. Sections were suspended in sterile Gey's balanced salt solution (GIBCO) with glucose (6.4 mg/ml) and separated with gentle perturbation. Slices were transferred to Millipore trans-well inserts (30 mm Millicell-CM, 0.4 mm pore membranes, Millipore, Bedford, MA). The transwell inserts were placed in 35 mm culture wells (Nalgene) containing 1 ml of growth medium and cultured at 37° C in a humidified 5%CO2/95% air incubator (Forma Scientific, Marietta OH). Growth medium consisted of Minimal essential medium-25 mM HEPES (50% vol), heat-inactivated horse serum (25% vol), and Hanks' balanced salt solution (Life Technologies, Rockville) (25% vol), supplemented with D-glucose (25.6 mg/ml) and glutamine (2 mM), final pH 7.2. Cultures were fed twice weekly for 14 days prior to treatment. Treatments were carried out for 7 days with a change of medium after 4 days . Statins (simvastatin (lactone) and fluvastatin from LKT Laboratories, St. Paul MN) were suspended in DMSO and diluted in growth medium before addition to culture. Control cultures contained DMSO at appropriate concentrations, in all cases less than 0.1%.

### Cortical neuron cultures

Cortical neuron cultures were prepared as previously described from E18 rats [15]. Cultures were plated on poly-D-lysine coated plates at a density of 106 cells/ml, and established for 2 weeks prior to experimental assessment. Plating medium consisted of minimal essential medium containing 10% fetal bovine serum and antibiotics (1% of penicillin G 104 U/ml, streptomycin 10 mg/ml, amphotericin B 25 ug/ml). Three hours after plating, medium was changed to culture medium consisting of Neurobasal medium with 1x B-27 supplement (Life Technologies, Rockville, MD).

### Schwann cell cultures

Schwann cell cultures were prepared as previously described from 1-day-old rat pups[16,17]. Cultures were established for 2 weeks prior to experimental assessment. Culture medium consisted of Neurobasal medium (GIBCO) with 1% fetal bovine serum (Hyclone, Logan UT) and glial cell line-derived neurotrophic factor (GDNF,) at 10 ug/ml. Schwann cells were plated into 96-well plates, using brief trypsinization, 24 h prior to experimental treatment.

### Immunochemistry and assessment of spinal motoneuron cultures

Cultures were fixed with 4% paraformaldehyde in 0.1 M Sorenson's buffered solution (pH 7.4) for 30 min at room temperature. Immunochemistry was performed as previously described [18]. Slices were permeabilized with methanol, 20 min at 4°C. Rinsing with Tris-buffered saline preceded blocking with 10% normal goat serum, 1 h at room temperature. Exposure to primary antibody (SMI-32, 1:8000) was at 4°C overnight. Signal amplification with Vectastain ABC (Vector, Burlingame, CA) preceded chromogenic development with diamino-benzidine (DAB) (Polyscience, Warrington, PA).

Spinal motor neurons were specifically identified in the organotypic slices based on cell size, morphology, location in the ventral horn, and dense staining with SMI-32. Motor neuron counts were carried out in a blinded fashion on 12–16 explants for each condition and confirmed by a second investigator.

Additional cultures and immunochemical studies were performed on organotypic slice cultures with 4 days of treatment to ascertain whether neuritic degeneration preceded motoneuron degeneration. For this, slices were placed onto collagen-coated transwell inserts, known to promote the outgrowth of neurites under appropriate conditions. Chromogenic development was performed using FITC-conjugated secondary antibody. Visualization of these slices was carried out using a Nikon Fluorescence microscope.

### Assessment of cortical neuron cultures and Schwann cell cultures

As a measure of statin cytotoxicity for cortical neurons and Schwann cells, ATP levels were measured as previously described [19] with minor modifications. Briefly, cells were plated into 96 well plates for 24 h prior to treatment with statins, vehicle control solution or medium control solution. Following 24 h of experimental treatment, measurement of ATP levels was performed using a commercially available luciferase-linked ATPase enzymatic assay (Vialight Plus, Cambrex).

### Statistical analysis

The number of spinal motor neurons in the slices of the experimental groups did not follow a normal distribution, for this reason median values are reported. For between group comparisons, data were transformed using a logarithmic function (log (N + 1)). Comparisons between groups were made on transformed data using a Chi-squared test with correction for multiple comparisons. 50% toxicity value for fluvastatin was estimated from data following a log-linear transformation of the data.

## Results

**Figure 1:**
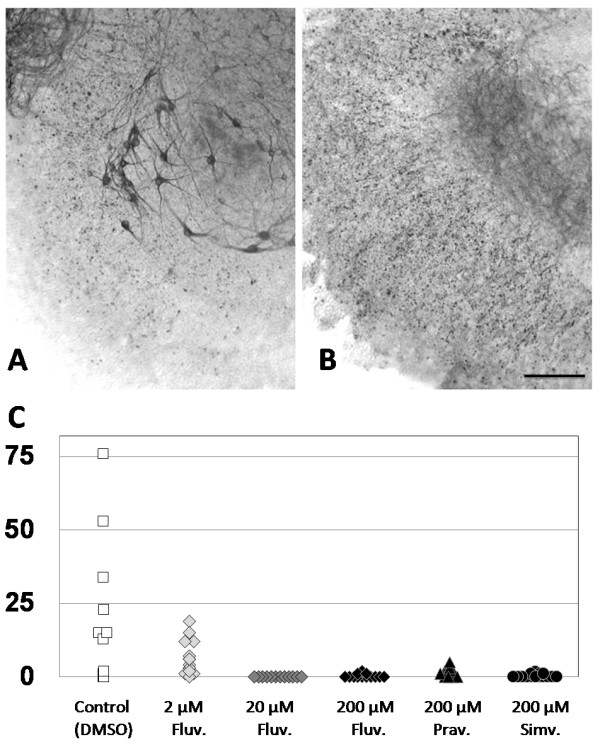
** Effect of statin exposure on motoneurons in organotypic spinal cord slices.** Organotypic spinal cord slices were cultured for 3 weeks with exposure to vehicle control solution (**A**) or 20 μM fluvastatin (**B**) occurring during the last week of culture. Cultures were stained with SMI32 antibodies developed with DAB as described in the text, and spinal motoneurons are immediately apparent in panel A, control cultures. A loss of spinal motoneurons is evident following treatment with fluvastatin, shown in panel B. Bar equals 300 μ. **C**) Quantitative assessment of statin effects on spinal motoneurons in organotypic slice cultures. Number of spinal motoneurons per slice for treatment with vehicle control solution or fluvastatin at 2 μM, 20 μM and 200 μM, pravastatin 200 μM and simvastatin 200 μM. All treatment groups demonstrated statistically significant decreases from control, after correction for multiple comparisons.

Three commonly used statins were tested for toxicity to spinal motoneurons in culture, these included fluvastatin, pravastatin, and simvastatin. As a group, the organotypic slice cultures retained normal conformational properties and were adherent to the membrane indicative of overall cellular integrity, consistent with observations of little or no toxicity of statins for glial cells. Spinal motor neurons were strikingly depleted or absent from cultures exposed to fluvastatin, compare Figure 1A and 1B.

Quantitation of spinal motoneurons showed that the median number per slice was 15 in the vehicle control group. The median number of motoneurons per slice was 0 in the 200 μM fluvastatin and simvastatin treatment groups, a significant decrease in motoneuron numbers (*p* < .001), Figure 1C. Treatment with 200 μM pravastatin yielded a median of 1 cell per slice, and also showed toxicity compared with control (*p* < .001). A limited dose–response curve for fluvastatin was obtained. Fluvastatin at 20 μM, was toxic for cultured motoneurons, median cells per slice was 0, (*p* < .001). Fluvastatin at 2 μM, was associated with partial toxicity, the median number of motoneurons of 5. This was significantly different from control, (*p* < .001) and from 20 μM and 200 μM fluvastatin, (*p* < .001). Replicate experiments showed analogous results.

**Figure 2:**
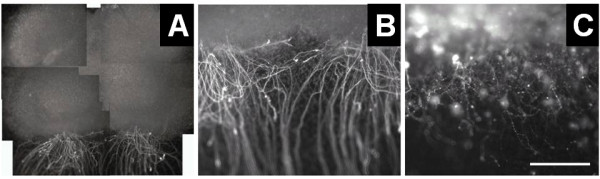
** Effect of statin exposure on neurite outgrowth.** Fluvastatin exposure for 96 h resulted in a marked degeneration of neurites growing out from organotypic spinal cord cultures, when plated on collagen coated membranes. Cultures were stained with SMI32 antibodies developed with FITC-conjugated secondary antibody as described in the text. Vigorous outgrowth of ventral horn neurites is apparent in panel **A**, montage view (bar equals 1 mm) and panel **B**, detail view (bar equals 500 μm). Neuritic degeneration is seen in panel C (bar equals 500 μm).

Further assessment was made using cultures with only 4 days of statin exposure. Control organotypic slice cultures grown on collagen coated membranes exhibited robust ventral neurite outgrowth after 2.5 weeks in culture. By contrast, organotypic slices exposed to 200 μM fluvastatin for 96 h demonstrated profound neuritic degeneration, Figure 2. Motoneuron staining, somewhat reduced, was still observable in these cultures.

**Figure 3:**
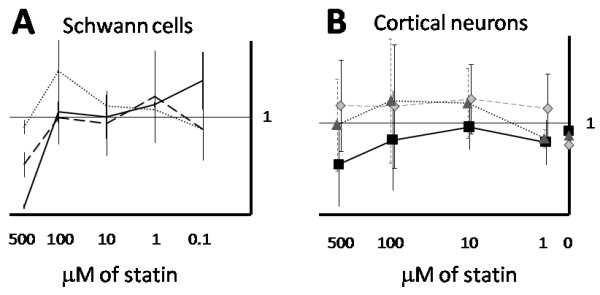
** Effect of statin exposure on primary Schwann cell and cortical neuron cultures. A**) Statin treatment of Schwann cells for 24 h did not result in significant toxicity except for very high concentrations of fluvastatin and simvastatin (500 μM). Three different statins are shown: fluvastatin, solid line; simvastatin, long dash; pravastatin, short dash. **B**) Statin treatment of cortical neurons for 24 h did not result in significant toxicity. Three different statins are shown: fluvastatin, solid line; lovastatin, long dash; pravastatin, short dash. Data are shown normalized to vehicle control, error bars indicate standard deviation from the mean.

In order to assess whether spinal motor neurons were unusual in exhibiting cytotoxic responses to statin exposure, we assessed the effects of statin treatment on cortical neuron cultures and primary cultures of Schwann cells. No potentially relevant cytotoxic effects were observed for these cell types, Figure 3.

## Discussion

In this study, we demonstrate that in vitro exposure of spinal motoneurons to selected statins, especially fluvastatin, resulted in marked cell loss. Shorter term exposures to fluvastatin resulted in neuritic degeneration. Other statins, specifically pravastatin and simvastatin, also exhibited toxic effects towards spinal motor neurons, but at much higher concentrations. The susceptibility of spinal motor neurons was in contrast to primary cultures of cortical neurons and peripheral nerve-derived Schwann cells which did not demonstrate significant deleterious changes. The results of this study provide evidence that specific pathophysiological mechanisms may underlie reports of neuromuscular disease exacerbation with statin exposure, however the clinical implications of this study remain to be determined.

There are multiple factors which might account for the differences between the clear in vitro toxicity reported here and the uncertainty surrounding observations of statin toxicity for spinal motor systems in clinical settings. Among the biological factors proposed to explain the wide clinical tolerability of statins are pharmacokinetics. Statins as a group are subject to high first-pass metabolism by the liver. This means that systemic levels are substantially below what might be predicted based on simple dilutional calculations. Statins do vary in the degree of first pass metabolism with the earlier-developed statins being more extensively metabolized [6]. Interestingly the low first-pass metabolism of cerivistatin, initially hailed as a breakthrough, may have contributed to the high-rate of serious adverse events [3]. In addition, most statins are hydrophobic, with several newer statins being especially so. Pravastatin is uniquely hydrophilic and likely requires distinct consideration when considering systemic effects. In our study, there was the suggestion that pravastatin may be somewhat less toxic to cultured spinal motoneurons that fluvastatin, however further studies are needed. Finally, compartmental pharmacokinetics may limit the effects of statins on spinal motor neurons. The extent to which specific statins attain significant concentrations in the spinal fluid is not well understood. Studies in laboratory animals have indicated that the permeability of lipophilic statins, simvastatin and lovastatin into rat brain is orders of magnitude greater than that of pravastatin [20]. Thus, there are multiple pharmacokinetic reasons why statins may exhibit lower in vivo than in vitro toxicity.

Pharmacokinetic data for fluvastatin from clinical trials of the drug, suggest that a more cautious interpretation may warranted. From the early pharmacokinetic studies in man, it is estimated that the peak daily serum levels of fluvastatin after a 40 mg dose is in the low single micromolar range [21]. The results of the fluvastatin dose response studies we report here provide evidence that the drug concentration necessary to produce 50% toxicity for spinal motoneurons in culture is less than 1 μM. Taken together, these data suggest that systemic levels of statins may approach levels that are toxic to spinal motoneurons and that particular mechanisms must account for the relatively infrequent occurrence of clinically demonstrable toxicity. Perhaps sequestration of spinal motoneurons in the spinal space is one such mechanism.

The pharmacokinetics of fluvastatinare are such that peak concentrations are present in the body for only a short period of time following single-dose administration [21]. For this reason, early fluvastatin treatment followed a twice-daily dosing regimen. This approach has subsequently been supplanted by the use of extended release formulations but for reasons that are not known, fluvastatin remains among the least commonly prescribed statins.

Based on these studies, it is not possible to specify a mechanism or mechanisms by which statins produce depletion of spinal motoneurons in culture. Intriguingly, somewhat analogous effects of statins have been reported in myotube cultures, where it was observed that fluvastatin and simvastatin exhibit clearly evident toxicity, the effects of pravastatin were apparently more mild [6]. Mechanisms by which statins have produced undesirable and desirable effects in other systems continue be explored. The effects of statins are widely acknowledged to be protean. Statins have profound impacts on inflammatory signaling pathways [22,23], are known to effect the linkage of G proteins to the various cell membranes [24,25], are thought to play a role in cancer signaling [26], have been shown to have direct actions on carnitine palmitoyl transferase localized to mitochondrial membranes [6], can disrupt oxidative phosphorylation and mitochondrial membrane potential [6] as well as interfering with cholesterol synthesis [27]. As an example of a one potential mechanism, statins have been shown to disrupt the attachment of Rho and Ras-family G proteins to cell membranes. It is interesting that one Ras-family protein, Rab5, is critically important for early neuronal endocytosis [28] and has been implicated in producing an ALS phenotype resulting from Alsin mutations [29]. The se animals may represent an especially attractive model system for further exploration of statin effects on motor neurons. Among these studies is a report that statins promote apoptosis of a glioma cell line mediated by ERK1/2 and AKT [25]. This is in contrast to our observations of robust resistance to statin effects in primary cultures of Schwann cells suggesting that important differences between normal and transformed glia that underlie statin effects on glioma cells. Thus, statins have a variety of effects on cell signaling. We here describe the observation of deleterious effects on the survival of spinal motor neurons in culture and because the mechanisms of statin effects on specific neuronal cell populations remain relatively obscure, additional study is warranted.

## Conclusions

In this study, we demonstrate that in vitro exposure of spinal motoneurons to selected statins, especially fluvastatin, resulted in marked cell loss. Shorter term exposures to fluvastatin resulted in neuritic degeneration. The results of this study provide evidence that specific pathophysiological mechanisms may underlie reports of neuromuscular disease exacerbation with statin exposure.

## Competing interests

The authors declare that they have no competing interests.

## Authors’ contributions

BBM contributed to the planning and conduct of all the experiments, statistical analysis, manuscript preparation and revisions. NH contributed to the planning of the experiments, the preparation of the cortical neuronal cultures and manuscript revisions. NM contributed to the planning of the experiments, the preparation and interpretation of the spinal motor neuron cultures and manuscript revisions. All authors read and approved the final manuscript.

## Pre-publication history

The pre-publication history for this paper can be accessed here:

http://www.biomedcentral.com/1471-2474/13/100/prepub
